# Meniscus repair via collagen matrix wrapping and bone marrow injection: clinical and biomolecular study

**DOI:** 10.1007/s00264-023-05711-2

**Published:** 2023-02-11

**Authors:** Paweł Bąkowski, Adam Aron Mieloch, Filip Porzucek, Monika Mańkowska, Kinga Ciemieniewska-Gorzela, Jakub Naczk, Tomasz Piontek, Jakub Dalibor Rybka

**Affiliations:** 1 Department of Orthopedic Surgery, Rehasport Clinic, Poznan, Poland; 2 Center for Advanced Technology, Adam Mickiewicz University in Poznan, Poznan, Poland; 3 Department of Spine Disorders and Pediatric Orthopedics, University of Medical Sciences, Poznan, Poland

**Keywords:** Knee, Ligaments, ACL; Knee, Meniscus; Biologic healing enhancement; Bone marrow aspirate concentrate; Cell/molecular biology; Meniscus repair, Collagen membrane, AMMR, Matrix wrapping

## Abstract

**Purpose:**

The functional outcomes of arthroscopic matrix-based meniscus repair (AMMR) in patients two and five years after the treatment clearly show that the use of the collagen matrix and bone marrow aspirate creates favorable biological conditions for meniscus healing. This study not only provides ten follow-up results but also investigates biomolecular mechanisms governing the regenerative process.

**Methods:**

Case series was based on data collected from patients who underwent AMMR procedure, starting with preoperatively through two-year and five-year till ten-year follow-up. The outcomes are presented as IKDC and the Lysholm subjective scores as well as the imaging results. Biomolecular investigation of the membranes utilized in the AMMR procedure include DNA content analysis, cell viability and proliferation study of bone marrow and bone marrow concentrate-derived cells, and cytokine array performed on monocytes cultured on the membranes.

**Conclusion:**

Data collected from patients who underwent AMMR procedure, starting with pre-operatively through two year and five year till ten year follow-up, indicate the possibility for long-term, stable meniscus preservation. Outcomes are manifested with a visible improvement of the IKDC and the Lysholm subjective scores as well as in the imaging results. The type of the meniscal tear or complexity of the knee injury (isolated AMMR vs. AMMR + ACL) did not affect the clinical outcomes. The study highlighted the role of the membrane in facilitating cell adhesion and proliferation. Additionally, several cytokines were selected as potentially crucial products of the membrane vs. monocyte interactions, driving the tissue regeneration and remodeling. Interestingly, thresholds of what constitutes a safe and well-decellularized membrane according to relevant literature have been significantly breached, but ultimately did not elicit detrimental side effects.

**Supplementary Information:**

The online version contains supplementary material available at 10.1007/s00264-023-05711-2.

## Introduction

Meniscus injury or meniscectomy decreases the meniscus function, followed by the initiation and the development of knee osteoarthritis. Therefore, the meniscus repair is recommended whenever possible [1]. One would expect that the ratio of meniscal repair has gradually increased. Indeed, multiple studies have clearly shown that the number of meniscus repairs is increasing; however, it was noticed that the meniscectomy is still frequent worldwide [2–4].

The success of the treatment of meniscal tears depends largely on the biological possibilities of healing of the damaged tissue and the so-called mechanical silence, which is a stable connection of the damaged parts of the meniscus. The healing processes after meniscal repair in the avascular zone are significantly restricted without an adequate blood supply, even when the meniscal repair technique can stabilize the tear. To enhance the healing after a meniscal repair, biological augmentation techniques appear to have significant potential, especially in the avascular zones. This includes the use of the growth factors, stem cells, platelet-rich plasma (PRP), introduction of a fibrin clot, and the use of mechanical stimulus [5]. Therefore, when the quality of the meniscus tissue and the type of tear allows for the application of stable sutures, but the prognosis of healing is doubtful, the biological support of meniscal healing is more than needed.

One of the advanced methods of biological support for meniscus healing is meniscus augmentation with a collagen membrane combined with the bone marrow aspirate injection. In 2012 a fully arthroscopic technique of treating the meniscal tears by suturing with the Fast-Fix sutures and wrapping in the collagen matrix (AMMR) was developed, followed by an injection of a liquid bone-marrow, collected from the tibial proximal epiphysis, into the area of the meniscal lesion [6]. The functional outcomes of this procedure in patients two and five years after the treatment clearly showed that the use of the collagen matrix and bone marrow aspirate created favorable biological conditions for meniscus healing [7, 8]. The purpose of the present work was to present a long-term, ten year follow-up of clinical results from a prospective consecutive case series of patients treated with a fully arthroscopic technique of collagen matrix-based meniscus repair (AMMR) combined with the injection of bone marrow aspirate into the area of the meniscal lesion.

Despite clinically proven efficacy, the interplay between molecular, cellular, and microenvironmental factors remains elusive. Without a thorough understanding of processes and interactions between a biomaterial, autologous components, and the injured tissue, meaningful progress in meniscus repair cannot be made. Therefore, we decided to support our clinical data with an investigation of biomolecular mechanisms relevant for the AMMR outcome. The experiments were designed to assess three major aspects: immune response to the biomaterial, cell survival, and proliferation of BM-derived cells, alterations in gene expression profiles of BM-derived cells when seeded on the membrane.

Monocytes play a central role in inflammation, which is one of the key aspects of tissue regeneration [9]. They can differentiate into one of the two macrophage subgroups: a pro-inflammatory (M1) or an anti-inflammatory/pro-resolving (M2). Based on this definition, M1 macrophages may initiate and sustain inflammatory responses, secrete pro-inflammatory cytokines, activate endothelial cells, and induce the recruitment of other immune cells into the inflamed tissue. Alternatively, M2 macrophages promote the resolution of inflammation, phagocytose apoptotic cells, drive collagen deposition, coordinate tissue integrity, and release anti-inflammatory mediators. In summary, macrophage polarization into functional phenotypes M1 or M2 is a key element of the immune response directing tissue remodeling and regeneration [10].

The membrane biocompatibility was determined by BM-derived cell adherence and viability on its surface. Subsequently, its potential for supporting differentiation into cartilage-specific cells was assessed by analyzing transcription profiles of genes involved in cell differentiation toward chondrocytes (e.g., SOX6 and SOX9) as well as those responsible for the production of metabolites characteristic for cartilage tissue — collagen type I, II, X, and aggrecan [11].

## Methods

### Study group

A total number of 53 patients meeting the inclusion criteria underwent the fully arthroscopic technique of the collagen matrix-based meniscus repair (AMMR) and the injection of bone marrow aspirate into the area of the meniscal lesion between April 2010 and November 2011. The two year follow-up period was achieved in 50 cases and a five year follow-up — by 44 patients [7, 8]. The present single-centre, retrospective study was conducted at Rehasport Clinic, Poznań, Poland, between March 2020 and June 2021 and included a total number of 23 patients who were available at ten years after the initial surgery and took part in the two year and five year follow-up evaluations. The study group consisted of six females (26%) and 17 males (74%), aged 36 ± 11 years old (18–50 years old), with a median BMI of 27 ± 3 (18–32) and a median lesion length of 30 ± 6 mm (15–40 mm). Sixteen patients underwent an isolated AMMR surgery and in seven cases AMMR was combined with the Anterior Cruciate Ligament reconstruction (AMMR + ACLR). The comprehensive data, including the demographics, of the patients participating in the ten year follow-up study are summarized in Supplementary Table 1.

### Surgical technique

Briefly, a routine diagnostic arthroscopy was performed to confirm the meniscus tear and the associated pathology. First, the loops of the threads were passed through the posterior root of the meniscus using the Accupass shuttle, and the looped ends were positioned facing the tibial side of the meniscus. The meniscus lesion was stabilized with the Fast-Fix sutures. In the next step, the collagen matrix (the usual size of 30 × 20 mm) was positioned around the stabilized meniscus with the porous part to the meniscal surface and fixed by Vicryl 1 arthroscopic simple knotted suture. Next, the GALL-BM11/10 equipment (Gallini Medical Devices, s.p.a. Italy) was used to collect approximately 5 ml of the liquid bone marrow from the proximal tibia. The bone marrow liquid was injected between the matrix and the meniscus, using a direct arthroscopic visualization with a “dry” arthroscopy technique. The surgery was completed by closing the wounds without drainage of the knee. No knee-stabilizing orthosis was used. The surgical technique was described in detail in previous studies [6].

### Evaluations

All patients were assessed post-operatively, ten years after the surgical treatment as previously described [7, 8]. The subjective outcomes were evaluated using an International Knee Documentation Committee knee ligament healing standard form (IKDC 2000) and the Lysholm knee scoring scale. The results of the IKDC Knee Index were classified as very good (90–100 points), good (76–89 points), sufficient (50–75 points), or insufficient (< 50 points). The results of the Lysholm scale were classified as excellent (90–100 points), very good (80–89 points), good (70–79 points), sufficient (60–69 points), or insufficient (< 60 points). The Whole Organ Magnetic Resonance Imaging Score (WORMS) was used to perform the multi-feature, whole-organ assessment of the knee using magnetic resonance images. WORMS examined a spectrum of osteoarthritis-related structural abnormalities including soft tissue, cartilage, and bone in the knee at various anatomical subregion locations.

### Statistical analysis

The statistical analysis has been performed using Statistica v. 7.1 software. The normality of the distribution has been verified using the Shapiro–Wilk test. There was no normal distribution, and hence, the quantitative variables have been presented using the median ± standard deviation. The Wilcoxon signed-rank test had been used to determine the significant differences between objective and subjective parameters. Statistical significance was set at *p* < 0.05. The statistical analyses of biomolecular studies were conducted using GraphPad Prism v. 8.1 software.

### Molecular analysis

Chondro-Gide (Geistlich Pharma AG) used in the study is a bilayer membrane composed of porcine collagen fibres type I and III. The top layer has a smooth, compact surface preventing cells from diffusion into joint space while the bottom layer consists of a porous collagen fibers matrix which facilitates cell attachment and proliferation.

The Angel System (Arthrex) was applied for the concentration of liquid bone marrow (BM) aspirated from the proximal tibia. Briefly, the system separates morphotic elements based on continuous centrifugation processes. Subsequently, the Angel utilizes three specific wavelengths of light to more precisely separate cell types after centrifugation. The Angel produces customized cellular products by changing haematocrit values.

The 100 µl of BM/BMC was poured on the bottom, porous layer of the Chondro-Gide membranes (0.25 cm^2^) and incubated for 1 h followed by the addition of cell culture medium (minimum essential medium (MEM) supplemented with 10% fetal bovine serum, penicillin/streptomycin 100 U/ml each, and L-glutamine to a final concentration of 4 mM). The membranes were cultured in 24-well non-adherent cell culture plates at 37 °C and 5% of CO_2_ for 21 days. An equal volume of BM/BMC was seeded on cell-adherent dishes and cultured under the same conditions to provide control for qPCR analysis. Cell viability was measured with CellTiter Glo 2.0 (Promega) luminescent assay by quantifying ATP, which indicates the presence of metabolically active cells. Briefly, the culture medium was removed from the membrane surface and a fresh medium mixed with CellTitter reagent was added. After the incubation, the solution was transferred to a 96-well white plate and luminescence level was measured with Tecan Infinite 200 Pro. The LIVE/DEAD® Viability/Cytotoxicity Kit (Invitrogen) and confocal microscope OLYMPUS FV120 were used for viability determination. The kit indicates intracellular esterase activity with green-fluorescent calcein-AM and stains cells with red-fluorescent ethidium homodimer-1 to indicate loss of plasma membrane integrity.

### DNA content analysis

DNA was isolated with a commercially available kit -Genomic Micro AX Tissue Gravity (A&A Biotechnology) according to the manufacturer’s protocol. Briefly, 10 mg of the membrane was precisely weighed and subjected to incubation in a lysis buffer with Proteinase K. Subsequently DNA was purified on the AXD column and eluted in a dedicated buffer. DNA content was measured with the use of the Qubit dsDNA BR kit and Qubit 4 device.

### Monocyte isolation and cytokine profiling

Monocytes were isolated from the whole blood of two healthy volunteers. First, the erythrocyte lysis step was applied (RBLC reagent, A&A Biotechnology) followed by Dynabeads® FlowComp™ Human CD14 kit (Thermo Fisher Scientific). The CD14 antibody was mixed with the sample and the CD14 + monocytes that bound the specific antibodies were captured by the beads and separated on a magnet. At the final step, the CD14 + monocytes were released from the beads by adding a release buffer. Subsequently, isolated monocytes were seeded on membranes and non-adherent cell culture plates (control group) and cultured for 21 days in DMEM medium supplemented with 10% ultra-low IgG bovine serum and penicillin/streptomycin 100 U/ml each. Culture media were harvested and cytokine presence was detected with a semi-quantitative antibody array consisting of 120 targets (ab193656, Abcam) according to the manufacturer protocol. Array membranes were analyzed with G:Box Chemi XX9 and GeneTools software (Syngene).

### Ethics

The study was performed with the approval of the local research ethics committee (Bioethics Committee at the Karol Marcinkowski Poznan University of Medical Sciences), in accordance with the Declaration of Helsinki, and all participants had provided their written informed consent of participation in this study.

## Results

The distribution of the IKDC and Lysholm subjective knee scores is presented in Table 1. A median value of the IKDC form for patients at ten year follow-up was excellent (< 90). It was statistically higher (*p* < 0.0001) than for the same groups tested pre-operatively, where a median value was lower than good (> 76). A similar correlation was noticed between the Lysholm score noted pre-operatively for all patients as well as for patients operated with AMMR technique solely (median score — sufficient) and at the ten year follow-up time (median score — excellent), with statistical importance of *p* < 0.0001. A median value of the Lysholm score before AMMR surgery combined with ACL reconstruction was good (> 76). However, it was statistically lower (*p* < 0.0001) than for the same group tested ten years after, where a median value was excellent. The differences in all parameters between patient groups operated with AMMR or AMMR + ACLR were significantly important (*p* > 0.5).

**Table 1:** The distribution of the IKDC and Lysholm subjective knee scores

Test	All patients	AMMR patients	AMMR + ACLR patients
IKDC preoperative	44 ± 14 (16–69)	44 ± 13 (16–64)	44 ± 15 (26–69)
IKDC 10-year FU	92 ± 10 (75–100)	89 ± 8 (75–100)	95 ± 15 (60–100)
Lysholm preoperative	68 ± 17 (29–94)	66 ± 17 (29–87)	78 ± 13 (51–100)
Lysholm 10-year FU	94 ± 8 (72–100)	92 ± 6 (80–100)	90 ± 11 (72–100)

Values are presented as median ± standard deviation. The minimum and maximum values are given in brackets. *AMMR* – isolated arthroscopic technique of the collagen matrix-based meniscus repair; *AMMR* + *ACLR* – AMMR combined with Anterior Cruciate Ligament reconstruction; *FU* – follow-up

**Fig. 1:**
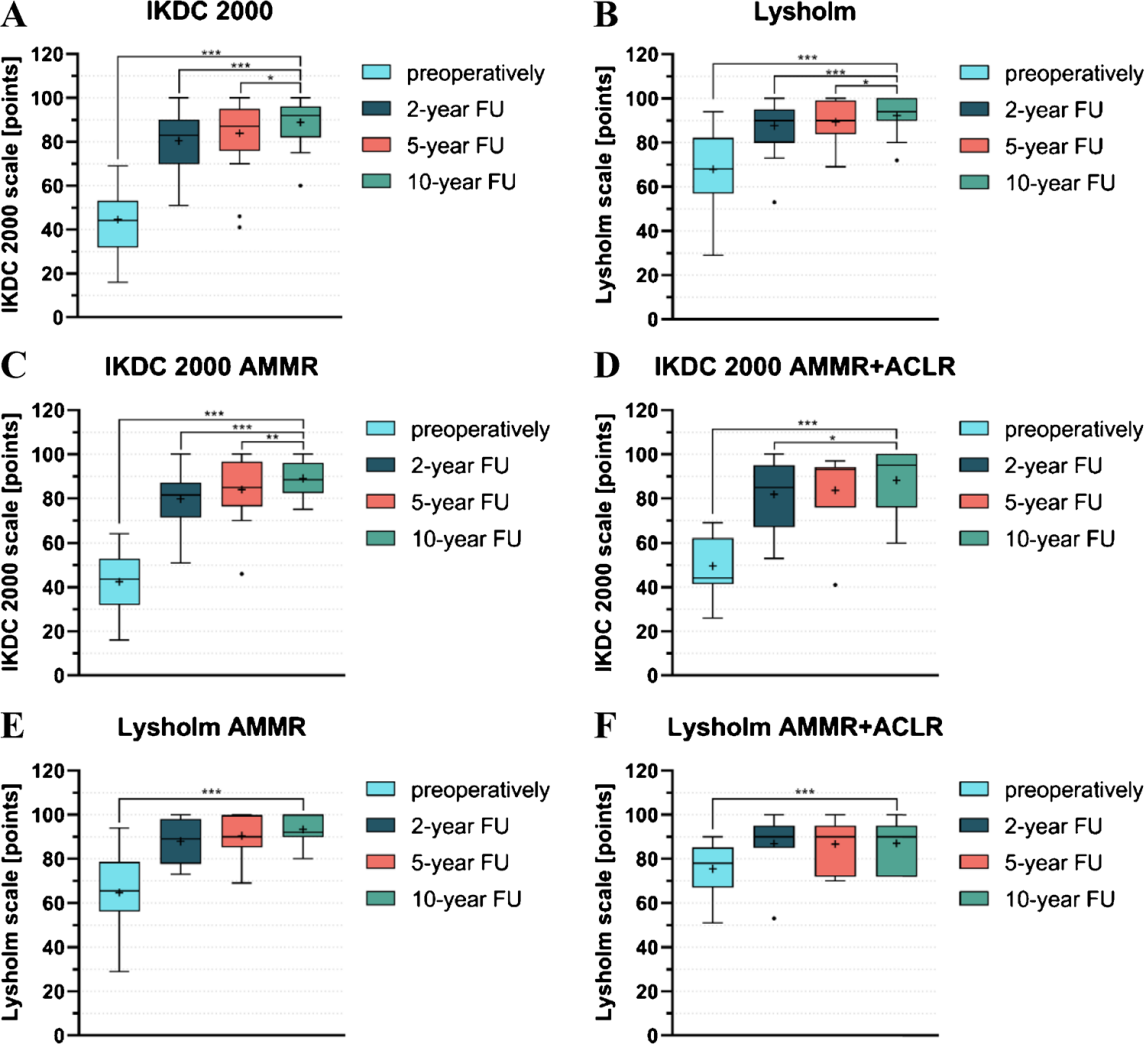
Improvement in the International Knee Documentation Committee (IKDC) (**A**, **C**, and **D**) and the Lysholm scores (**B**, **E**, and **F**) from the pre-operative level to 2 years, 5 years, and 10 years of follow-up. AMMR stands for patients treated with arthroscopic matrix-based meniscus repair, while ACLR denotes anterior cruciate ligament reconstruction *p < 0.05 **p < 0.01 ***p < 0.001

The scores of both IKDC and Lysholm increased in all patients over time relative to the pre-operative measurement (Fig. 1). While there was a significant difference between pre- and ten year post-operative scores (*p* < 0.001) as well as two year and five year post-operative scores (*p* < 0.001), the difference between scores at two year and ten year follow-up was lower, but still significant (*p* = 0.012 for IKDC and *p* = 0.013). To our best knowledge, out of 53 cases who were initially enrolled in the study, four were considered as failures. Two patients within the first year post-op required medical intervention and eventually underwent partial meniscectomy [7]. Another two underwent arthroscopic debridement for persistent knee pain and swelling following their AMMR within two to five years after the surgery [8].

The scores of the IKDC increased in all subgroups (Fig. 1A, C, D) over time, relative to the pre-operative measurement. The IKDC scores for patients five years and ten years after the AMMR combined with ACLR showed no statistically significant differences (Fig. 1C, D). The most prominent increase in the Lyscolm scores in both subgroups overtime was observed after two years of the AMMR or AMMR + ACLR surgery (Fig. 1E, F). In the subsequent follow-up studies, the scores plateaued.

The overall results of the Whole-Organ Magnetic Resonance Imaging Score indicated a statistically significant decrease when compared to the five year follow-up (*p* < 0.01) in all patients, as shown in Table 2. However, such differences were not observed when both subgroups were analyzed separately. Moreover, the difference in the post-operative MRI results for AMMR and AMMR + ACLR groups was not significant at the ten year follow-up study. The difference between the groups was only significant at a five year follow-up.

**Table 2:** The overall scores for Whole-Organ Magnetic Resonance Imaging Score (WORMS)

	All patients	AMMR	AMMR + ACLR	*p* value
WORMS 2-year FU	6.9 ± 5.0	5.0 ± 4.2	7.5 ± 5.0	n.s
WORMS 5-year FU	11.1 ± 9.6	6.0 ± 7.2	13.8 ± 11.0	*p* = 0.005
WORMS 10-year FU	8.0 ± 12.0	6.3 ± 2.9	12.3 ± 14.4	n.s

Values are presented as median ± standard deviation. *AMMR* – isolated arthroscopic technique of the collagen matrix-based meniscus repair; *AMMR* + *ACLR* – AMMR combined with Anterior Cruciate Ligament reconstruction; *FU* – follow-up

### DNA content analysis

**Fig. 2:**
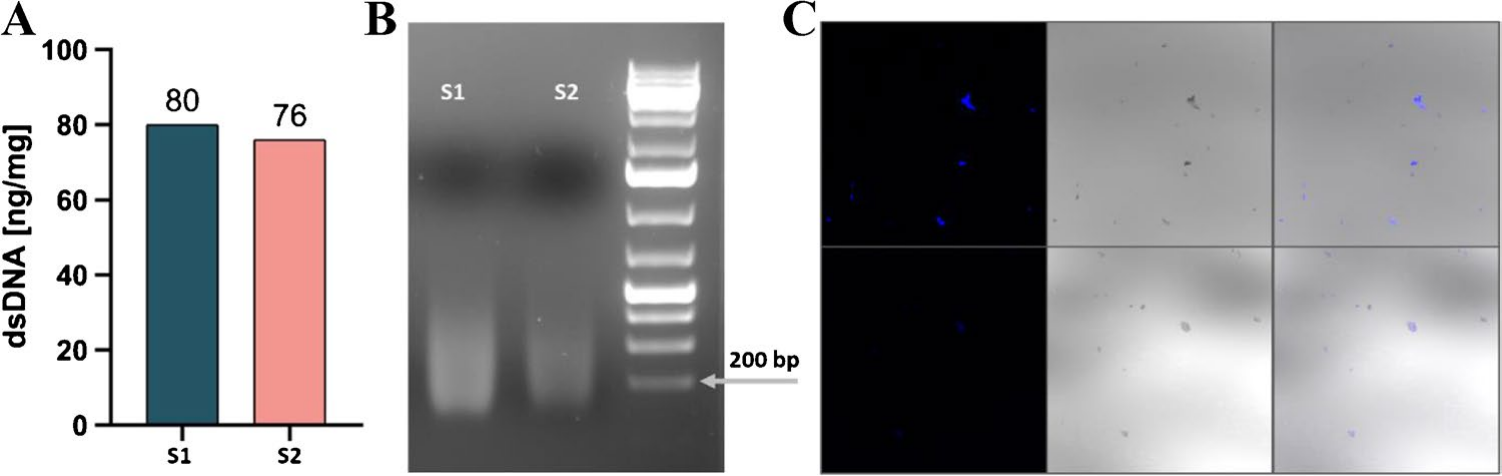
DNA quantitative and qualitative analysis. **A** dsDNA content measure with Qubit dsDNA BR kit. **B** Agarose-gel electrophoretic separation of the isolated DNA. **C** Membranes stained with Hoechst 33,342 dye, visualized with a confocal microscope. The blue color indicates nuclear material

As the literature indicates [12], leftover DNA content from decellularized animal tissues is not only an indirect marker of the decellularization efficacy but may also elicit an adverse immune response. A set of three criteria is proposed within relevant literature to render an ECM-based scaffold safe for human use: dsDNA content < 50 ng/mg of dry weight, the length of DNA fragments < 200 bp, and lack of visible nuclear material within the sample. As demonstrated in Fig. 2, all three criteria have been breached by the ChondroGide membrane.

### Cell viability and proliferation on the membrane surface

**Fig. 3:**
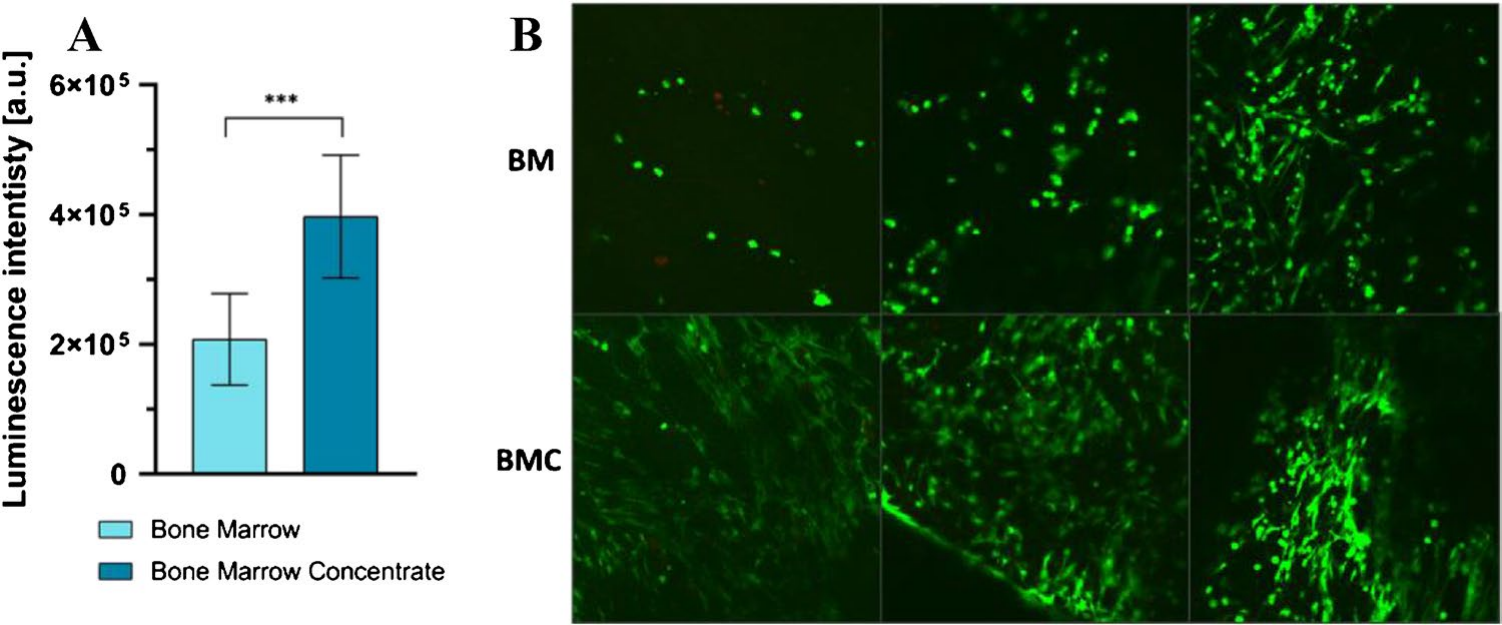
Cell viability and proliferation. **A** CellTiter Glo 2.0 assay. Statistical analysis: Welch’s *t*-test (*p* < 0.05). *p* = 0.0006. **B** Confocal imaging of LIVE/DEAD assay. Green dye – live cells; red dye – dead cells. For both assays, cells were cultured for 4 weeks

To assess the biocompatibility of the ChondroGide and cell delivery efficacy, bone marrow (BM) and bone marrow concentrate (BMC) derived from the same patients were seeded on the membrane surface and cultured for 21 days. As expected, the ATP metabolism assay showed a significant increase in cell quantity on membranes seeded with BMC compared to BM-seeded ones (Fig. 3A). After LIVE/DEAD staining (live and dead cells stained green and red, respectively), green-dyed cells were mostly observed on both BM- and BMC-treated membranes with the overall signal significantly stronger in the latter. Only sparse dead (red) cells were visible. Cells seeded on BM-treated membrane characterized with round-shaped morphology while images of BM-treated membrane revealed spindle-shaped cells which suggests their more efficient better attachment to the ChondroGide surface (Fig. 3B).

### Cytokine profile

Relative cytokine levels produced by monocytes/macrophages growing on a membrane and cultured on non-adherent plastic plates were compared. Differences between those were recognized as significant only if the same pattern was observed in samples collected from both studied donors. Importantly, this method provides semi-quantitative results and should be considered as a candidate selection tool. Nonetheless, obtained data suggest that monocytes seeded on the membrane were slightly stimulated towards the M2 macrophage phenotype; thus, the membrane is supporting anti-inflammatory response and tissue regeneration. Based on these results, a more detailed, quantitative analysis would be a noteworthy step forward (Table 3).

**Table 3:** Selected cytokine production levels and their role

Cytokine	Control	Membrane	Role	References
*MCP-1/CCL2*	** + + + + **	** + + + + **	- Pro-inflammatory - Secreted by monocytes - Responsible for M1 macrophage recruitment to inflammation site	[13, 14]
*MDC/CCL22*	** + + + **	** + + **	- Anti-inflammatory - Synthesized by monocyte-derived alternative (M2) macrophages - Receptor is expressed in regulatory T cells and Th2 cells	[15]
*MIP-1 alpha*	** + + **	** + **	- Pro-inflammatory - Chemokine secreted by macrophages - Responsible for recruiting inflammatory cells, wound healing, inhibition of stem cells, and maintaining effector immune response - Produced by cells during infection or inflammation	[16]
*MIP-1 beta*	** + + **	** + + **	- Pro-inflammatory - Produced by monocytes - Plays a major role in the recruitment of leukocytes to sites of infection - Modulates the production of cytokines by T helper (Th) cells	[17]
*Eotaxin*	** + + **	** + + **	- Produced by monocytes - Coordinates the recruitment of inflammatory cells, eosinophils in particular	[18]
*HGF*	** + **	** + **	- Anti-inflammatory - Promotes the transition to M2 macrophage - Facilitates muscle regeneration	[19]
*NT-3*	** + **	** + **	- Anti-inflammatory - Secreted by macrophages - Peptide growth factors - Promotes neuron survival and regeneration - Participate healing mechanisms and osteogenic differentiation	[20, 21]
*sTNFRI*	** + **	** + **	- Receptor for TNF-alpha pro-inflammatory cytokine	
*TIMP-2*	** + + + **	** + + **	- Pro-inflammatory - Inhibitor of the matrix metalloproteinases - Modulates cell proliferation, apoptosis, differentiation, and angiogenesis	[22]
*IL-6R*	** + + **	** + + **	- Receptor for IL-6	
*IL-6*	**-**	** + **	- Pro-inflammatory - Enhances the development of an M2 macrophages	[23]
*IL-8*	** + + + + **	** + + + + **	- Anti-inflammatory - Secreted by M2 macrophages	[10]
*IL-10*	**-**	** + **	- Anti-inflammatory - Secreted by M2 macrophages	[24]
*IL-15*	**-**	** + **	- Pro-inflammatory - Produced by hematopoietic progenitors, bone marrow stromal cells, macrophages	[25]
*uPAR*	** + + **	** + **	- Pro-inflammatory - Supports extracellular matrix degradation and regulates cell migration, adhesion, and proliferation - Secreted by M1 macrophages - Acts at several levels during an inflammatory response as a central regulator of macrophage, matrix remodeling, and adhesion	[26, 27]
*CTACK/CCL27*	** + **	** + + **	- Anti-inflammatory - Cutaneous T cell-attracting chemokine - Participates in wound repair by recruiting T cells, accelerates skin regeneration	[28]
*GRO*	**-**	** + + **	- Anti-inflammatory - Secreted by M2 macrophages	[29]
*AgRP*	**-**	** + **	- AgRP mRNA expression in rodents is increased in models of acute and chronic inflammation	[30]
*Acrp30*	**-**	** + **	- Anti-inflammatory - Suppresses M1 macrophage activation and promotes M2 macrophage proliferation - Downregulates pro-inflammatory cytokines (e.g., TNF-α, MCP-1, IL-6) in macrophages	[31]

Human Cytokine Antibody Array of 120 targets, from which 19 cytokines presented the same pattern between both donors

## Discussion

Understandably, saving the meniscus is an overarching goal of the current meniscus treatments. With recent advancements in surgical techniques, biomaterials, and cell-based tissue engineering, new therapeutic options are on the not too distant horizon [32–35]. This study brings forward the benefits of utilizing AMMR in combination with autologous bone marrow injection, and highlights biomolecular mechanism contributing to the favourable clinical outcomes.

Bone marrow-derived cells have been previously used to augment the regenerative potential of injured menisci [36]. First clinical attempt to implement autologous BM MSC cells in combination with collagen membrane was carried out by Whitehouse and colleagues [37]. In that study five patients received treatment for avascular region tear in order to prevent meniscectomy. In three of them, within two year follow-up clinical improvement in knee function was observed, confirmed with MRI scans, while the remaining two required meniscectomies due to recurring tear or non-healing tear. Drawback of this method, compared to our AMMR technique, is a need for previous in vitro cell culturing which delays the procedure. On the other hand, recent clinical trials were conducted by Olivos-Meza et al. [38], in which acellular polyurethane scaffold or polyurethane scaffold enriched with MSC were implanted. Despite the overall positive outcome of both procedures followed at 12 months post-op, the addition of MSC did not show any advantageous effect.

According to meta-analysis studies, pooled failure rate of surgical meniscus repair oscillates around 19 to 23% when studies with a minimum follow-up of five years are considered [39]. Most of the treatment failures (64%) occurred within the first two years, 23% within two to five post-operative years, after five years of surgery only 13% [39]. In reference to this data AMMR approach seems to be highly successful with the failure rate at the two year follow-up of 3.7% and pooled, ten year failure of 7.5%.

Data collected from patients who underwent AMMR procedure, starting with preoperatively through two year and five year till ten year follow-up, indicate the possibility for a long-term, stable meniscus preservation. Outcomes are manifested with a visible improvement of the IKDC and the Lysholm subjective scores as well as in the imaging results. The type of the meniscal tear or complexity of the knee injury (isolated AMMR vs. AMMR + ACL) did not affect the clinical outcomes. Moreover, the molecular tests of the collagen membranes used in AMMR, carried out in vitro, proved a good survival and viability of the bone marrow-derived cells seeded on the membrane suggesting its beneficial effect on the healing process. Additionally, anti-inflammatory cytokines’ presence on the membrane seeded with monocytes indicates its immunomodulatory properties which may positively modulate tissue regeneration. Encouraged by the promising results of our 10-year follow-up, in vitro studies were performed to elucidate basic molecular mechanisms governing the regenerative process, with hopes to improve the outcome of AMMR technique even further. We have concluded that the collagen membrane facilitates BM-derived cells’ adhesion, growth, and proliferation. We cannot unequivocally state which type of cells adheres to the membrane; however, based on the available literature and cell morphology, we assume that mesenchymal stem cells are the dominant population. With the use of freshly isolated human monocytes, we have confirmed that the membrane itself exhibits immunomodulatory properties, which may contribute significantly to tissue regeneration and remodeling. Additionally, obtained results provided evidence on concentrated bone marrow superiority over the freshly aspirated bone marrow in the in vitro setting.

Recently some concern has arisen on the safety of blood component administration in the course of meniscal tear repairs due to possible blood-induced joint damage [40]. Our study provides data supporting the positive effect of BM application on meniscus healing as well as on overall knee joint functionality. Also, we are aware that neither autologous BM nor BMC are standardized products. Therefore, patient-to-patient variability has to be taken into account while drawing conclusions. Another limitation of this study stems from only one type of collagen membrane used for AMMR procedure and in vitro. Results may vary for collagen membranes from different producers. Importantly, BMC has been only utilized for biomolecular analysis, and further studies are required to confirm its superior properties in comparison to BM. Additionally, as mentioned in the “Results” section, cytokine analysis was semi-quantitative and performed on two donors, highlighting cytokines of interest for further studies, rather than providing a definitive pathway of alterations.

### Supplementary Information

Below is the link to the electronic supplementary material.

Supplementary file1 (DOCX 23 KB)

## Data Availability

All data are available under request.
