# Supplementary material for: Meniscus repair via collagen matrix wrapping and bone marrow injection: clinical and biomolecular study
Source: Int Orthop. 2023 Feb 11;47(10):2409–17. doi: 10.1007/s00264-023-05711-2 (PMC10522727; doi:10.1007/s00264-023-05711-2)
Supplement: Supplementary file 1 — Supplementary file1 (DOCX 23 KB) [file 264_2023_5711_MOESM1_ESM.docx]

**Meniscus repair via collagen matrix wrapping and bone marrow injection: clinical and biomolecular study.**

**Authors:** Paweł Bąkowski^1^, Filip Porzucek^2^, Adam Aron Mieloch^2^, Monika Mańkowska-Woźniak^2^, Jakub Dalibor Rybka^2^, Tomasz Piontek^1,3^.

**Affiliations:**

^1^ Department of Orthopedic Surgery, Rehasport Clinic, Poznan, Poland

^2^ Center for Advanced Technology, Adam Mickiewicz University in Poznan, Poland

^3^ Department of Spine Disorders and Pediatric Orthopedics, University of Medical Sciences, Poznan, Poland

**Supplementary data**

**Supplementary table 1. Summary of the demographic data.**

Abbreviations: BMI - body mass index; F - female; FU - follow-up; M - male.

| ***No.*** | **Patient no in 2-year FU ^9^** | **Sex** | **Age (years)** | **BMI** | **Lesion size (mm)** | **Surgery** |
| --- | --- | --- | --- | --- | --- | --- |
| *1* | 4 | M | 18 | 24 | 30 | AMMR + ACLR |
| *2* | 9 | F | 46 | 27 | 25 | AMMR + ACLR |
| *3* | 11 | F | 46 | 22 | 20 | AMMR |
| *4* | 12 | M | 44 | 29 | 25 | AMMR |
| *5* | 13 | M | 50 | 32 | 30 | AMMR |
| *6* | 15 | M | 31 | 27 | 40 | AMMR + ACLR |
| *7* | 19 | M | 35 | 30 | 30 | AMMR |
| *8* | 20 | M | 23 | 26 | 30 | AMMR |
| *9* | 21 | M | 26 | 27 | 40 | AMMR + ACLR |
| *10* | 22 | M | 50 | 27 | 30 | AMMR |
| *11* | 28 | F | 20 | 21 | 20 | AMMR |
| *12* | 32 | M | 33 | 25 | 15 | AMMR + ACLR |
| *13* | 33 | M | 39 | 28 | 25 | AMMR |
| *14* | 34 | M | 48 | 28 | 30 | AMMR + ACLR |
| *15* | 35 | F | 37 | 18 | 30 | AMMR |
| *16* | 38 | F | 36 | 21 | 30 | AMMR |
| *17* | 39 | M | 18 | 26 | 35 | AMMR |
| *18* | 40 | M | 41 | 27 | 35 | AMMR |
| *19* | 42 | M | 40 | 27 | 30 | AMMR + ACLR |
| *20* | 43 | F | 49 | 23 | 30 | AMMR |
| *21* | 44 | M | 19 | 27 | 30 | AMMR |
| *22* | 46 | M | 19 | 29 | 30 | AMMR |
| *23* | 48 | M | 28 | 22 | 30 | AMMR |
